# Detection of chikungunya virus in saliva and urine

**DOI:** 10.1186/s12985-016-0556-9

**Published:** 2016-06-16

**Authors:** Didier Musso, Anita Teissier, Eline Rouault, Sylviane Teururai, Jean-Jacques de Pina, Tu-Xuan Nhan

**Affiliations:** Laboratoire de biologie médicale, Institut Louis Malardé, PO Box 30, 98713 Papeete, Tahiti French Polynesia; Pôle de recherche et de veille sur les maladies infectieuses émergentes, Institut Louis Malardé, Tahiti, French Polynesia

**Keywords:** Chikungunya, CHIKV, PCR, Saliva, French Polynesia, Arbovirus, Urine

## Abstract

**Background:**

Saliva and urine have been used for arthropod-borne viruses molecular detection but not yet for chikungunya virus (CHIKV). We investigated the use of saliva and urine for molecular detection of CHIKV during the French Polynesian outbreak.

**Methods:**

During the French Polynesian chikungunya outbreak (2014–2015), we collected the same day blood and saliva samples from 60 patients with probable chikungunya (47 during the 1st week post symptoms onset and 13 after), urine was available for 39 of them. All samples were tested using a CHIKV reverse-transcription PCR.

**Results:**

Forty eight patients had confirmed chikungunya. For confirmed chikungunya presenting during the 1st week post symptoms onset, CHIKV RNA was detected from 86.1 % (31/36) of blood, 58.3 % (21/36) of saliva and 8.3 % (2/24) of urine. Detection rate of CHIKV RNA was significantly higher in blood compared to saliva. For confirmed chikungunya presenting after the 1st week post symptoms onset, CHIKV RNA was detected from 8.3 % (1/12) of blood, 8.3 % (1/12) of saliva and 0 % (0/8) of urine.

**Conclusions:**

In contrast to Zika virus (ZIKV), saliva did not increased the detection rate of CHIKV RNA during the 1st week post symptoms onset. In contrast to ZIKV, dengue virus and West Nile virus, urine did not enlarged the window of detection of CHIKV RNA after the 1st week post symptoms onset. Saliva can be used for molecular detection of CHIKV during the 1st week post symptoms onset only if blood is impossible to collect but with a lower sensitivity compared to blood.

## Background

French Polynesia (FP), south Pacific, is a high endemic area for arthropod-borne viruses (arboviruses) [1]. Until 2013, dengue virus (DENV) (arbovirus of the genus *Flavivirus*) has been the only arbovirus detected in FP, causing multiple outbreaks from the 1960s [2]. Zika virus (ZIKV) (arbovirus of the genus *Flavivirus*), emerged in FP in 2013 causing an outbreak from October 2013 to April 2014 [3, 4]. Chikungunya virus (CHIKV) (arbovirus of the genus *Alphavirus*) [5], emerged in the Pacific in 2011 and subsequently spread throughout the region [6]. A CHIKV outbreak occurred in FP from October 2014 to March 2015 [7] with an estimate of 66,000 cases (about 25 % of the population) [6].

During the FP ZIKV outbreak, we developed molecular ZIKV detection on saliva as an alternative sample to blood [8]. The ability to detect ZIKV was higher in saliva compared to blood at the acute phase of the illness. This protocol was of particular interest when blood was difficult to collect, especially for children and neonates. For ZIKV [9–11], DENV [12, 13] or West Nile virus (WNV) [14, 15], the use of urine for molecular diagnosis can enlarge the window of detection of these arboviruses.

As alternative sample to blood have been reported to increase the detection rate or to enlarge the window of detection of arboviruses, we investigated the use of saliva and urine for CHIKV molecular detection during the FP CHIKV outbreak.

## Methods

The study was conducted in FP over a 4 months period from November 2014 to February 2015, during the FP CHIKV outbreak.

Patients presenting in our laboratory with a medical prescription for chikungunya diagnosis were asked to provide, in addition to blood, saliva and urine sample. A standardized medical questionnaire form was available for all patients, it included the numbers of days after symptoms onset and the main clinical symptoms.

After informed written consent was obtained, blood was collected by venous puncture, saliva was collected with dry cotton swabs without transport media (N° 150C, Copan, Brescia, Italy), and urine was collected on sterile containers without additives. We included in the study patients with possible chikungunya infection with both blood and saliva, and if possible urine, samples collected at the same time.

Case definition for possible and confirmed CHIKV were defined according the World Health Organization [16] and European Center for Diseases Prevention and Control [17] criteria. Possible cases were patient with clinical criteria of chikungunya (acute onset of fever > 38.5 °C and severe arthralgia/arthritis) not explained by other medical conditions; confirmed cases were patient meeting the laboratory criteria [virus isolation or presence of viral RNA by Reverse Transcription (RT)-PCR] presence of virus specific IgM antibodies in single serum sample collected in acute or convalescent stage, four-fold increase in IgG values in sample collected at least 3 weeks apart), irrespective of the clinical presentation.

According to the French recommendations [18] blood samples were tested by RT-PCR for patients collected from days 1 to 4 post symptoms onset (PSO), by RT-PCR and serology for patients collected from days 5 to 7 PSO and by serology for patients collected after day 7 PSO. In order to compare the use of RT-PCR in saliva and urine to blood, all samples were tested by CHIKV RT-PCR, including blood samples collected after day 7 PSO.

For molecular detection of CHIKV, RNA extraction was performed using the NucliSENS® easyMAG® System (BioMérieux) according to manufacturer’s recommendations. Two hundred μl of serum or 500 μl of urine were added to 2 ml of lysis buffer, oral swabs were first vortexed in 2 ml of lysis buffer, then all were eluted by elution buffer and 5 μl of extracted RNA was used for amplification on a CFX96 Touch™ Real-Time PCR detection System (Biorad) using primers/probe amplification sets specific for CHIKV as previously reported [7, 19].

Immunoglobulin M (IgM) detection was performed using Novalisa Chikungunya IgG/IgM μ-capture ELISA kit (NovaTec Immundiagnostica GmbH, Germany) according to the recommendations of the supplier.

## Results

Sixty patients were included in the study, of them 48 had confirmed chikungunya (36 presenting during the 1st week PSO and 12 after), 32 infections have been confirmed by detection of CHIKV RNA and 16 by detection of specific IgM antibodies against CHIKV (Table 1).

**Table 1:** Patients included in the study

	Possible chikungunya	Confirmed chikungunya
Week 1 post symptoms onset	47	36/47 (76.6 %)
After week 1 post symptoms onset	13	12/13 (92.3 %)
Total	60	48/60 (80.0 %)

Blood and saliva were available from all patients. Urine was available from 39 patients (32 from confirmed chikungunya).

Saliva and urines RT-PCR results for patients with confirmed chikungunya are reported in Tables 2 and 3 respectively.

**Table 2:** Saliva RT-PCR results for confirmed chikungunya (48 patients)

		Saliva RT-PCR	
		Positive	Negative
Week 1 post symptoms onset (*n* = 36)	Positive blood RT-PCR	21	10
	Positive blood IgM	0	5
(*n* = 12)	Positive blood RT-PCR	1	0
	Positive blood IgM	0	11

**Table 3:** Urines RT-PCR results for confirmed chikungunya (32 patients)

		Urine RT-PCR	
		Positive	Negative
Week 1 post symptoms onset (*n* = 24)	Positive blood RT-PCR	2	17
	Positive blood IgM	0	5
After week 1 post symptoms onset (*n* = 8)	Positive blood RT-PCR	0	0
	Positive blood IgM	0	8

For confirmed chikungunya presenting during the 1st week PSO, CHIKV RNA was detected from 86.1 % (31/36) of blood, 58.3 % (21/36) of saliva and 8.3 % (2/24) of urine. Detection rate was significantly higher in blood compared to saliva (Fisher's exact test, *p* = 0.008), it was not significantly higher in blood compared to urine (Fisher's exact test, *p* = 1) but the number of patients was too small to observe a significant difference.

Samples testing positive in urine by RT-PCR were collected on days 3 and 5 PSO.

For confirmed chikungunya presenting after the 1st week PSO, CHIKV RNA was detected from 8.3 % (1/12) of blood, 8.3 % (1/12) of saliva and 0 % (0/8) of the urine. Detection rate was not significantly different when we compared blood to saliva (Fisher's exact test, *p* = 0.08) and urine (Fisher's exact test, *p* = 0.08).

**Fig. 1:**
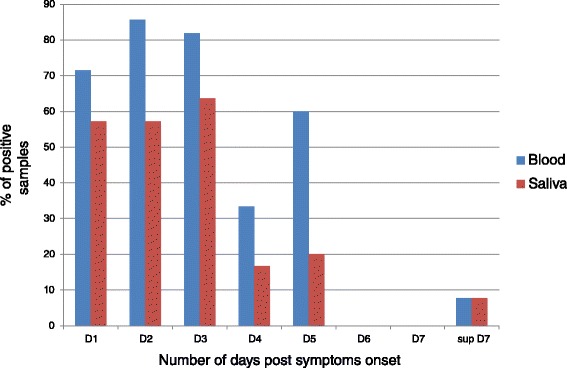
Proportion of positive samples according to the number of days after symptoms onset for the 60 patients with both blood (blue) and saliva (red) samples tested by ZIKV RT-PCR

The proportion of positive RT-PCR saliva samples according to the number of days PSO is reported in Fig. 1.

None of the 12 patients who tested negative by CHIKV-RT-PCR in blood was positive in saliva and/or urine.

## Discussion

Urine and saliva as alternative samples to blood have been used successfully for molecular arbovirus detection [8–15, 20–26].

During the FP ZIKV outbreak, the use of saliva increased the detection rate of ZIKV RNA at the acute phase of the disease [8], saliva was also used to detect ZIKV from an Australian traveller returning from Indonesia [20]. Detection of dengue virus (DENV) RNA in saliva was reported [12, 21]. CHIKV RNA and infectious CHIKV in saliva were found only in patients with gingivorrhagia [22]. In our study, none of the 22 patients with saliva testing positive for CHIKV reported hemorrhagic manifestations, nevertheless we cannot exclude that subclinical gingivorrhagia were responsible of positivity of saliva due to contamination by viremic blood. In contrast to ZIKV [8], CHIKV detection in saliva did not increased the rate of molecular detection of CHIKV. As saliva was positive in 58.3 % of confirmed chikungunya presenting during the 1st week PSO, saliva can be of interest for molecular CHIKV detection only if blood samples are impossible to collect, especially if collected from the first 3 days PSO.

Arbovirus detection in urine was reported at the acute and late phase of dengue [12, 13, 23], WNV [14, 15], CHIKV [26] and ZIKV infection [9–11, 24, 25]; and during Japanese encephalitis [27] and yellow fever [28] infections. The main interest of urine was to enlarge the window of detection for DENV [12, 13], WNV [14, 15] and ZIKV [9–11, 25] after the acute phase. CHIKV was detected in urine of experimentally infected mice at day 30 post infection, after CHIKV clearance in blood [29]. In our study, the two urines samples testing positive for CHIKV were collected during the 1st week PSO and all samples collected after tested negative.

## Conclusions

According to our results, saliva cannot replace blood for molecular detection of CHIKV. Saliva specimen can be used only during the 1st week PSO if blood is impossible to collect and with the restriction that the sensitivity is low. In contrast to DENV, WNV and ZIKV, urine does not enlarge the window of detection of CHIKV RNA. If saliva and urine are of great interest for the detection of ZIKV RNA, blood remains the sample of choice for CHIKV, DENV and WNV RNA detection.

These results should be confirmed on a larger study.

## Abbreviations

CHKV, chikungunya virus; DENV, dengue virus; FP, French Polynesia; Ig, Immunoglobulin; PSO, Post symptoms onset; RT-PCR, reverse transcription-Polymerase chain reaction; WNV, West Nile virus; ZIKV, Zika virus.
