# Erratum to: Detection of chikungunya virus in saliva and urine

**DOI:** 10.1186/s12985-016-0570-y

**Published:** 2016-07-04

**Authors:** Didier Musso, Anita Teissier, Eline Rouault, Sylviane Teururai, Jean-Jacques de Pina, Tu-Xuan Nhan

**Affiliations:** Laboratoire de biologie médicale, Institut Louis Malardé, PO Box 3098713, Papeete, Tahiti French Polynesia; Pôle de recherche et de veille sur les maladies infectieuses émergentes, Institut Louis Malardé, Papeete, Tahiti French Polynesia

## Erratum

Upon publication, it was noticed that in the Acknowledgements section of the original article [1], ‘Chikungunya’ was mistakenly written as ‘Zika’. The acknowledgements have now been corrected below:

We are grateful to the technical team of the medical analysis laboratory of Institut Louis Malardé for their contribution in performing the Chikungunya virus molecular testing.

In addition, the city ‘Papeete’ was missing from affiliation 2 in the original article. This has now been corrected.
